# The linear mitochondrial genome of commensal hydroid *Eutima japonica* (*Cnidaria*, *Hydrozoa*, *Eirenidae*)

**DOI:** 10.1080/23802359.2021.1899869

**Published:** 2021-03-19

**Authors:** Jung Soo Seo, Hey-Jin Eom, Jae-Kwon Cho, Hyun-Sil Kang, Jae-Sung Rhee

**Affiliations:** a Southeast Sea Fisheries Research Institute, National Institute of Fisheries Science, Tongyeong, South Korea; b Department of Marine Science, College of Natural Sciences, Incheon National University, Incheon, South Korea; c Research Institute of Basic Sciences, Incheon National University, Incheon, South Korea

**Keywords:** Commensal hydroid, *Eutima japonica*, Leptothecata, linear mitogenome, phylogeny

## Abstract

Here, we present the whole mitochondrial genome of commensal hydroid *Eutima japonica* McCrady 1859 (family Eirinidae); this is the first specimen of the family to have its mitogenome sequenced. The linear mitogenome is 15,315 bp in length and consists of 13 protein-coding genes (PCGs), large and small ribosomal subunits (rRNA), methionine and tryptophan transfer RNA (*tRNA*) genes (trnM and trnW), and a partial copy of cytochrome oxidase subunit I (*cox1*) pseudogene, as is typical for the class Hydrozoa. Nucleotide sequences of two *cox1* genes at two ends of the linear mitogenome form a part of inverted terminal repeat. The overall genomic structure and gene arrangement of 13 PCGs were identical to the reported mitochondrial genomes of hydrozoans, except for the positions of two *tRNA* genes. Phylogenetic analysis of *E. japonica* 13 PCGs and other cnidarians recovers a closest relationship with the derived cluster of two hydrozoans, *Laomedea flexuosa* and *Obelia longissimi* within Leptothecata.

Bivalve-inhabiting hydrozoans have lifestyles from simple epibiosis to mutualistic or commensalistic symbiosis with numerous aquatic animals (Piraino et al. 1994; Gili and Hughes 1995). Two genera of hydroids, *Eutima* McCrady, 1859 and *Eugymnanthea* Palombi, 1935 (e.g. *Eutima japonica*, *E. sapinhoa*, and *Eugymnanthea japonica*) as members in the family Eirenidae are generally observed inhabiting the bivalves *via* direct attachment to soft body parts (e.g. mantle) (Kubota 1992, 2012). Since controversy has occurred on the origin of Eirenidae and evolutionary relationships in hydrozoans, molecular phylogenetic approach has been conducted using several marker genes or transcriptome information (Leclère et al. 2009; Maronna et al. 2016; Kubota and Collins 2017; Kayal et al. 2018). Since partial genomic information such (e.g. *16S rRNA*, *18S rRNA*, and *COI* genes) has been available in *Eutima* and *Eugymnanthea* yet, their complete mitogenomes will provide a robust resource to understand phylogenetic relationship and evolutionary history of the bivalve-inhabiting Eirinidae species.

A specimen of *E. japonica* was collected in the mantle cavity of the Pacific oyster *Crassostrea gigas* from Southeast Sea in Korea (34°50′16.1′′N, 128°14′30.8′′W). The voucher specimen was registered in the Southeast Sea Fisheries Research Institute (Species ID: Cnidaria-01; Specimen ID: NIFS-SSFRI-01). Genomic DNA was isolated using a QIAamp DNA Blood Mini kit (Qiagen, Hilden, Germany). A genomic library was constructed using a MGIEasy DNA library prep kit (MGI, Shenzhen, China) by MOAGEN (Pusan, Busan, South Korea), based on the manufacturer’s instructions. The quality of the library was checked using the Agilent 2100 bioanalyzer (Agilent Technologies, Santa Clara, CA) and raw reads were obtained by MGI MGISEQ-2000 sequencing platform (paired-end, 150 bp read length, MGI). After the trimming process of 3′ adapter on the paired-end reads using Cutadapt version 1.9 (Martin 2011), the mitochondrial genome of *E. japonica* was recovered by direct mapping to the hydrozoan mitogenomes using Geneious version 11.1.3 (Kearse et al. 2012). Mitogenome feature and gene annotation were performed using MITOS2 (Bernt et al. 2013) and tRNAscan-SE version 2.0 (Lowe and Eddy 1997), and finally, the annotated gene was carefully confirmed using NCBI-BLAST (http://blast.ncbi.nlm.nih.gov).

In this study, the complete linear mitochondrial genome of *E. japonica* was assembled and annotated as the first mitogenome of a member in the family Eirinidae. The mitogenome for *E. japonica* (GenBank accession no. MW066348) was 15,315 bp long and had a GC content of 32.8% (A: 35.5%; T: 31.7%; G: 12.7%; C: 20.1%). The *E. japonica* mitochondrion contained 13 PCGs, 22 *tRNA* genes (trnM and trnW), two *rRNA* genes, and a partial copy of *cox1* pseudogene (Figure 1((A)). The order of 13 PCGs was identical to the reported 34 mitochondrial genomes of hydrozoans, except for the positions of two *tRNA* genes. The *trnM* gene was located between the *cox3* and *ND2*, and the position of *trnW* gene was between *cox2* and *ATP8*. Genomic positions of trnM and trnW were analyzed between *cox1* pseudogene-*16S rRNA* and *16S rRNA*-*cox2* in the genus *Hydra* (Kayal and Lavrov 2008; Pan et al. 2014).

**Figure 1.**
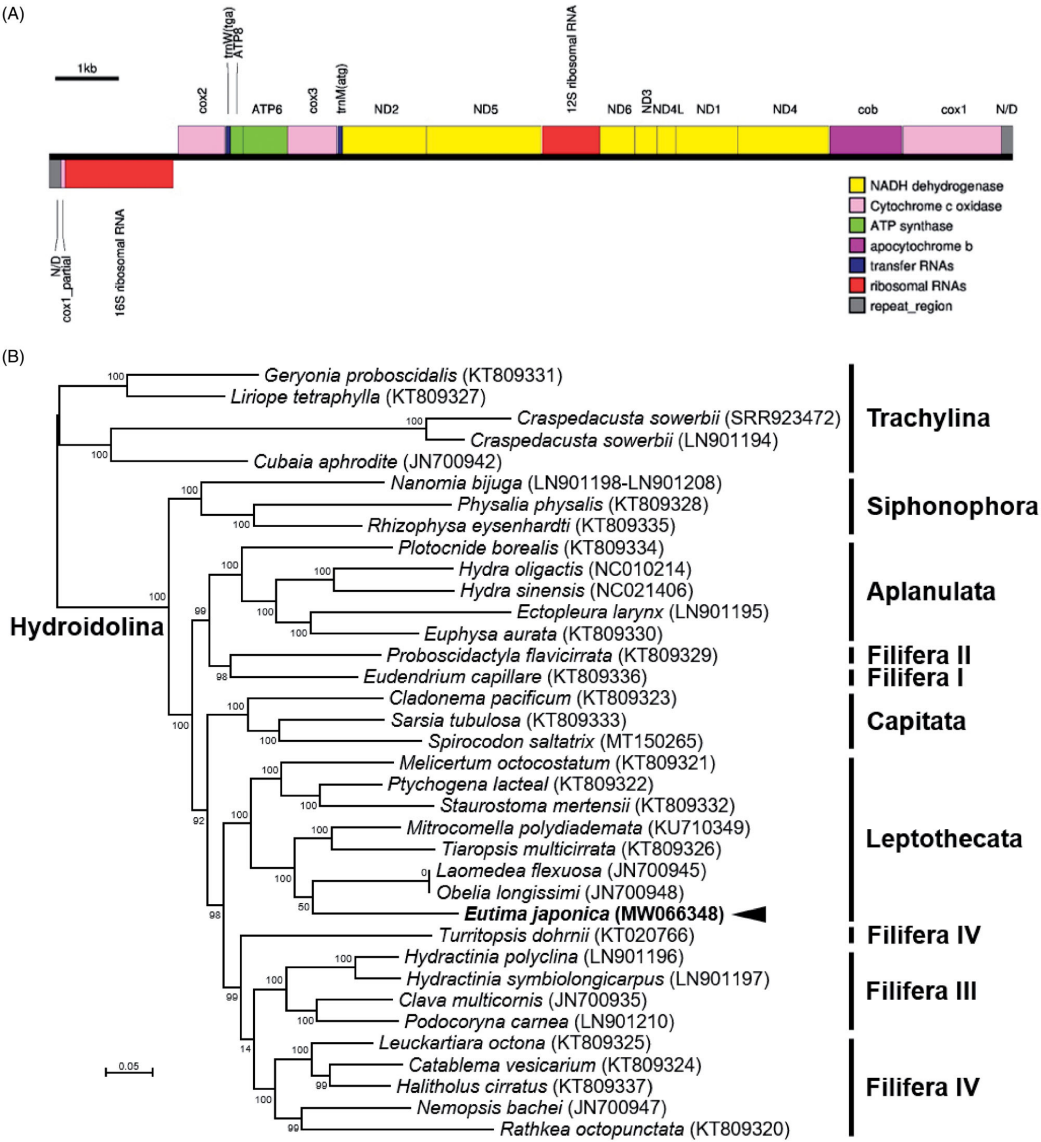
(A) Schematic diagram for the linear genomic structure of *E. japonica* mitogenome. (B) Maximum-likelihood (ML) phylogeny of 36 hydrozoans including *E. japonica* based on the concatenated nucleotide sequences of entire protein-coding genes (PCGs). Numbers at nodes represent ML bootstrap percentages (1000 replicates). DDBJ/EMBL/Genbank accession numbers for published sequences are incorporated. The black arrow indicates the *Eutima sp.* analyzed in this study.

A maximum-likelihood phylogenetic hypothesis was established using sequence data from the concatenated set of 13 PCGs of *E. japonica* mitogenome with including 35 published hydrozoan mitogenomes. JModelTest version 2.1.10 (Darriba et al. 2012) was used to select the best substitution model and a substitution model (HKY + G + I) was applied to perform a maximum-likelihood (ML) method in the PhyML version 2.4.5 (Guindon and Gascuel 2003) with 1000 bootstrap replicates. Hydrozoa consists of two main clades, Trachylina and Hydroidolina (Aplanulata, Capitata *s*.*s*., Filifera I–IV, Leptothecata, Siphonophorae) that comprises almost all hydrozoans except for Limnomedusae, which is part of Trachylina (Kayal et al. 2015). Based on the monophyly of Hydroidolina and Trachylina (Kayal et al. 2015), five members of Trachylina were set as an outgroup. Phylogenetic analysis using mitogenomic data resolved a close relationship of *E. japonica* with the cluster of Leptothecata species (Figure 1(B)).

## Data Availability

BioProject, SRA, and BioSample accession numbers are https://www.ncbi.nlm.ni h.gov/bioproject/PRJNA684984, https://www.ncbi.nlm.nih.gov/sra/SRR13249693, and https://www.ncbi.nlm.nih.gov/biosample/SAMN17073642, respectively. The data that support the findings of this study are openly available in the National Center for Biotechnology Information (NCBI) at https://www.ncbi.nlm.nih.gov, accession number MW066348.

## References

[CIT0001] Bernt A, Donath A, Jühling F, Externbrink F, Florentz C, Fritzsch G, Pütz J, Middendorf M, Stadler PF. 2013. MITOS: improved de novo metazoan mitochondrial genome annotation. Mol Phylogenet Evol. 69(2):313–319. 22982435 10.1016/j.ympev.2012.08.023

[CIT0002] Darriba D, Taboada GL, Doallo R, Posada D. 2012. jModelTest 2: more models, new heuristics and parallel computing. Nat Methods. 9(8):772. 10.1038/nmeth.2109 PMC4594756 22847109

[CIT0003] Gili JM, Hughes RG. 1995. The ecology of marine benthic hydroids. Oceanogr Mar Biol Annu Rev. 33:351–426.

[CIT0004] Guindon S, Gascuel O. 2003. A simple, fast, and accurate algorithm to estimate large phylogenies by maximum likelihood. Syst Biol. 52(5):696–704. 14530136 10.1080/10635150390235520

[CIT0005] Kayal E, Bentlage B, Cartwright P, Yanagihara AA, Lindsay DJ, Hopcroft RR, Collins AG. 2015. Phylogenetic analysis of higher-level relationships within Hydroidolina (Cnidaria: Hydrozoa) using mitochondrial genome data and insight into their mitochondrial transcription. PeerJ. 3:e1403. 26618080 10.7717/peerj.1403 PMC4655093

[CIT0006] Kayal E, Bentlage B, Pankey MS, Ohdera AH, Medina M, Plachetzki DC, Collins AG, Ryan JF. 2018. Phylogenomics provides a robust topology of the major cnidarian lineages and insights on the origins of key organismal traits. BMC Evol Biol. 18:68.

[CIT0007] Kayal E, Lavrov DV. 2008. The mitochondrial genome of *Hydra oligactis* (Cnidaria, Hydrozoa) sheds new light on animal mtDNA evolution and cnidarian phylogeny. Gene. 410(1):177–186. 18222615 10.1016/j.gene.2007.12.002

[CIT0008] Kearse M, Moir R, Wilson A, Stones-Havas S, Cheung M, Sturrock S, Buxton S, Cooper A, Markowitz S, Duran C, et al. 2012. Geneious Basic: an integrated and extendable desktop software platform for the organization and analysis of sequence data. Bioinformatics. 28(12):1647–1649. 22543367 10.1093/bioinformatics/bts199 PMC3371832

[CIT0009] Kubota S. 1992. Four bivalve-inhabiting hydrozoans in Japan differing in range and host preference. p. Sci Mar 56:149–159.

[CIT0010] Kubota S. 2012. The life cycle of a bivalve-inhabiting hydrozoan, *Eutima sapinhoa* (Cnidaria, Hydrozoa), from Florida, USA. Biogeography. 14:87–91.

[CIT0011] Kubota S, Collins A. 2017. A single origin of bivalve-inhabiting hydrozoans (Cnidaria, Hydrozoa, Leptomedusae) in the family Eirenidae based on an analysis of *16S rRNA* gene. Biogeography. 19:75–79.

[CIT0012] Leclère C, Schuchert P, Cruaud C, Couloux A, Manuel M. 2009. Molecular phylogenetics of Thecata (Hydrozoa, Cnidaria) reveals long-term maintenance of life history traits despite high frequency of recent character changes. Syst Biol. 58(5):509–526. 20525605 10.1093/sysbio/syp044

[CIT0013] Lowe TM, Eddy SR. 1997. tRNAscan-SE: a program for improved detection of transfer *RNA* genes in genomic sequence. Nucleic Acids Res. 25(5):955–964. 9023104 10.1093/nar/25.5.955 PMC146525

[CIT0014] Maronna MM, Miranda TP, Peña Cantero ÁL, Barbeitos MS, Marques AC. 2016. Towards a phylogenetic classification of Leptothecata (Cnidaria, Hydrozoa). Sci Rep. 6:18075–23. 26821567 10.1038/srep18075 PMC4731775

[CIT0015] Martin M. 2011. Cutadapt removes adapter sequences from highthroughput sequencing reads. EMBnet J. 17(1):10–12.

[CIT0016] Pan H, Qian X, Li P, Li X, Wang A. 2014. The complete mitochondrial genome of Chinese green hydra, *Hydra sinensis* (Hydroida: Hydridae). Mitochondrial DNA. 25(1):44–45. 23638951 10.3109/19401736.2013.782017

[CIT0017] Piraino S, Todaro C, Geraci S, Boero F. 1994. Ecology of the bivalve-inhabiting hydroid *Eugymnanthea inquilina* in the coastal sounds of Taranto (Ionian Sea, SE Italy. Mar Biol. 118(4):695–703.

